# The Relationship Between the Burnout Syndrome Dimensions and Body Mass Index as a Moderator Variable on Obese Managers in the Mexican *Maquiladora* Industry

**DOI:** 10.3389/fpsyg.2021.540426

**Published:** 2021-02-04

**Authors:** Oziely Armenta-Hernández, Aidé Maldonado-Macías, María del Rocío Camacho-Alamilla, Miguel Ángel Serrano-Rosa, Yolanda Angélica Baez-Lopez, Cesar Omar Balderrama-Armendariz

**Affiliations:** ^1^Electric and Computing Engineering Department, Universidad Autónoma de Ciudad Juárez (Autonomous University of Ciudad Juarez), Ciudad Juarez, Mexico; ^2^Industrial Engineering and Manufacturing, Universidad Autonoma de Ciudad Juárez (Autonomous University of Ciudad Juarez, Ciudad Juarez, Mexico; ^3^Department of Psychobiology, Universidad de Valencia (University of Valencia), Valencia, Spain; ^4^Facultad de Arquitectura, Ingeniería y Diseño, Universidad Autónoma de Baja California (Autonomous University of Baja California), Baja California, Mexico; ^5^Department of Industrial Design, Universidad Autónoma de Ciudad Juárez (Autonomous University of Ciudad Juarez), Ciudad Juarez, Mexico

**Keywords:** obesity, Burnout Syndrome (BS), professional efficacy, middle and senior management, *maquiladora* industry, body mass index

## Abstract

Burnout syndrome (BS) and obesity are two growing conditions that affect employees’ health and company productivity. Recently, several studies have pointed to a possible relationship between both phenomena. However, such a relationship has not been clearly defined. This research analyzes the relationship between BS dimensions and body mass index (BMI), the latter being treated as a moderator variable among obese senior and middle managers in the Mexican *maquiladora* industry through a structural equation model. A total of 361 senior and middle managers (124 of them classified as obese under the World Health Organization’s criteria) completed both the Maslach Burnout Inventory-General Survey [with emotional exhaustion (EE), cynicism, and professional efficacy (PE) as subscale dimensions] and a sociodemographic questionnaire (which included BMI). The results showed a statistically significant relationship between EE and PE (*P* < 0.001; β = -0.320), with BMI acting as a moderator variable. The results showed that when BMI increases as a moderator variable, the strength of the relationship between EE and PE also changes. For example, although PE had a negative value of 0.14 before the moderator effect, the value increased up to 0.32 when the BMI was factored into the relationship. Therefore, *maquiladora* industries are being advised to increase their investments on the identification and prevention of employees’ EE and obesity. Such interventions would promote a better quality of life and could prevent economic losses resulting from poor employee performance.

## Introduction

There are two large health problems in Mexico: high levels of work stress, whose chronic form is known as Burnout Syndrome (BS), and an increasing number of obese people. The literature suggests that both problems could be related to each other. This paper aims to explore such a relationship more deeply. According to surveys conducted by the Mexican Social Security Institute (I), Mexico features a high rate of incidence of work stress as 75% of Mexican employees experience it at work, whereas in China (73%) and the United States percentages drop to 73 and 59%, respectively (Estrés Laboral, 2013). Moreover, results from a recent study conducted by the *Universidad Autónoma de Nuevo León* (Autonomous University of Nuevo Leon) among 500 Mexican professionals showed all the study subjects to feature a certain degree of BS and more than 60% of them to experience it in high levels (CIDICS 2018). Finally, because both obesity and work stress are related to cardiovascular and metabolic diseases, they lead directly to high rates of premature death or diseases, which takes a toll on the productivity of organizations.

The BS is an affective state characterized by feelings of emotional exhaustion (EE), physical fatigue, and cognitive weariness, all of which lead to a depletion of energetic resources as a result of the constant exposure to chronic work and life stress (Armon et al., 2010; Tavella et al., 2020). Additionally, BS can have a negative impact on cognitive performance and mood affecting employees’ productivity and performance (Penz et al., 2018). The condition consists of three dimensions: EE, cynicism, and professional efficacy (PE). The first one (EE) is characterized by weariness and fatigue, which can be either physical or mental. Cynicism, on the other hand, refers to an attitude of indifference, personal devaluation, and detachment from work, and defensiveness toward exacting job demands (Moreno-Jiménez et al., 2001; Cohan and Davenport, 2018; Tavella et al., 2020). Finally, PE is associated with poor work performance and lack of productivity (Maslach and Jackson, 1981; Cohan and Davenport, 2018). Consequently, the BS has adverse effects on employees’ wellbeing and performance (Bianchi et al., 2015b). A national survey conducted by an insurance company in the United States found that 25% of workers perceive their job as their main stressor and that for seven out of ten employees; work is a common cause of health problems and decreased productivity (Hudson, 2005; Bianchi et al., 2015a). Furthermore, a study conducted by Global HR Consulting in the departments of engineering, manufacturing, and marketing for a wide range of companies revealed that one out of seven managers had lost one or more staff members as result of BS and that a third of the sample had seen a decline in productivity (van den Berg et al., 2011).

Currently, the three main causes for productivity decrease are lack of control over one’s job, insufficient skills, and high work demands (Srivastava and Barmola, 2011; Aranda et al., 2014). Additionally, some authors have related these causes to other mental conditions as depression (Bianchi et al., 2015a; Parker and Tavella, 2021). Regarding the BS, low productivity is a result of physical and EE (Borritz et al., 2010; Tavella et al., 2020), which, in turn, contribute to high levels of employee absenteeism (Zhang and Feng, 2011). In addition, employee attrition (Van Bogaert et al., 2010; Schaufeli et al., 2009; Tong et al., 2020) earlier departure from work (Bosqued, 2008), poor performance, production errors (Ross, 1993), and quality issues (Srivastava and Barmola, 2011; Dai et al., 2021). Work relationships can be deteriorated because of cynicism (Rubio, 2003; Ahola et al., 2012; Tavella et al., 2020). According to the American Institute of Stress, all these consequences cost the US industry more than $300 billion dollars a year. Additionally, in industrial employees under 45 years old, the risk of mortality has increased by 26% because of exhaustion, by 29% due to cynicism, and by 22% because of lack of PE (Schaufeli et al., 2009). In addition, diverse occupations worldwide have been affected by BS (Bouza et al., 2020). Clearly, the BS is affecting productivity.

The second problem, already mentioned, is obesity. The Mexican population shows high rates of obesity, which is currently considered a national health priority since more than 70% of the adult population is overweight. Among individuals in middle and senior management positions, specifically, obesity is on the rise. The World Health Organization (WHO) defines obesity as a condition characterized by abnormal or excessive fat accumulation (Dávila-Torres et al., 2015) resulting from several and complex factors and an imbalance between the calories consumed and those expended (Proper et al., 2013; Fox et al., 2019). Body mass index (BMI), which considers a person’s weight (kg) and height (m), is normally used as an obesity/overweight indicator. In this regard, the WHO describes a person as overweight when his/her BMI is equal to or higher than 25 kg/m^2^; obese, when the BMI is equal to or higher than 30 kg/m^2^; and featuring normal weight, when the BMI ranges between 18.5 and 24.9 kg/m^2^ (World Health Organization, 2020).

The effects of obesity in working environments are productivity loss, compensations for disability and extra deployment of support services for sufferers (Finkelstein et al., 2010; Bomberg et al., 2017). Consequently, the economic losses derived from health issues among full-time workers in the United States reach an estimate of 73.1 billion dollars each year (Erazo, 2012). Moreover, obesity is related to feelings of work insecurity among workers (Han et al., 2011), idleness (Luckhaupt et al., 2014), a hostile work environment (Hellerstedt and Jeffery, 1997), perceptions of high psychological job demands (Blandina-Fernández and González-Jaimes, 2014), and scarce or deficient managerial support (Honkonen et al., 2006). Similarly, other studies have aimed to analyze the effects of little physical activity, which is typical of the new sedentary jobs (Eberly and Feldman, 2010; Moueleu Ngalagou et al., 2019), the long work shifts (Charles et al., 2009), and the prolonged working hours (Luckhaupt et al., 2014). Among the consequences found are an increase in somnolence (Kivimäki et al., 2006) and alterations in employees’ eating habits (Berset et al., 2011).

As can be seen, two of industrialized society’s most important problems coexist in Mexico, which makes this country a special place to study the relationship between both phenomena. Specifically, in Mexico, an important portion of the industrial sector focuses on export manufacturing. This sector is known as the *maquiladora* industry and consists of 5,171 manufacturing companies, which employ 2,730,816 workers, billing around $7,233.37 million US dollars yearly and making up 60% of the nation’s total exporting goods (INEGI, 2019). The Mexican state of Chihuahua alone generates 13.6% of the Mexican manufacturing, transnational industry’s entire income. Within Chihuahua, Ciudad Juarez is one of the ten most important industrial cities in Mexico and the most important United States-Mexico border city in terms of industrial development as it employs 279,900 workers across its approximately 324 different manufacturing companies. Additionally, Ciudad Juarez ranks first in industrial employment *per capita* as well as in exports. The city is also responsible for the creation of 20% of the nation’s industrial jobs (INEGI, 2019), which makes up about 14.2% of the industrial workforce in Mexico. Furthermore, Ciudad Juarez can reach the highest rates of production as well as the highest number and variety of industrial job positions nationwide. Finally, although the maquiladora production is exported mainly to the United States, other countries such as China and Korea are also active participants in this economic activity.

Such level of growth in the maquiladora industry has entailed high mental job demands and highly stressful work environments, which can increase the incidence of the BS among employees. Managers in the maquiladora industry regularly work long shifts in sedentary work conditions, which force them to adopt straining postures (Valadez-Torres et al., 2017; Armenta-Hernandez et al., 2020). Furthermore, the *maquiladoras*’ multicultural work environment places considerable mental burdens on them and demands additional job skills for which employees’ mental resources may be insufficient, all this resulting in BS. In addition, company managers are constantly engaged in complex decision-making processes inside contexts of uncertainty; where their very permanence on their jobs is at times just as uncertain. Such work conditions can affect the company’s organizational environment (Salgado and Mejía, 2008; Dyrbye et al., 2020). On the other hand, the long hours (up to 36 h in a row) that managers must sometimes work often compromise their eating habits and can, in turn, cause obesity (Montiel et al., 2014; Sirén et al., 2018). Lastly, one of the factors affecting managers the most is the constant need to interact with the people they oversee while trying to meet their superiors’ demands (Guerrero and Vicente, 2001; Macias-Velasquez et al., 2019; Armenta-Hernandez et al., 2020). As was mentioned before, the maquiladora industry is the main source of employment and foreign investment in Mexico, and because of their key role in it, middle and senior managers are exposed to highly stressful circumstances.

## Theoretical Framework: Relationships Among BS Dimensions and BMI

The initiative to study the relationship between BS and obesity is an attempt to increase the understanding of both conditions as worldwide problems since they both involve psychophysiological responses that cause negative changes in individuals’ behaviors (De Vriendt et al., 2009). This paper focuses on the BS; specifically on the mediating role that BMI plays in the relationships among the BS dimensions centering in detail in that one between EE and PE. The rationale of the analysis understands the employees’ performance as a dependent variable affecting their subjective involvement. Thus, employees’ performance and productivity depend on their skills and knowledge, emotional and physical variables as well; that is, the feeling of PE is a variable that we consider fundamental to understand managers’ involvement, performance, and productivity. Additionally, this variable can be influenced by the emotional state of workers (reflected in EE and cynicism). Accordingly, physical variables such as BMI can influence PE perception, which reflects a sedentary and unhealthy lifestyle among obese managers. This interdisciplinary and comprehensive approach aims to explore the complexity of these employees’ behavior. This behavior depends not only on psychosocial variables but also on physical ones as BMI, which could be just the tip of the iceberg to increasing knowledge of this lifestyle.

Although the BS is studied as a whole, several studies have approached its dimensions and the relationships among them by questioning their dependence or independence (Shirom et al., 2006). Thus, EE and cynicism (two central dimensions of the BS) are strongly associated to each other, while PE has weaker associations with the other dimensions (Kim and Ji, 2009). In this sense, the associations among the three dimensions could reflect the order of appearance. Leiter and Maslach (1988) suggested that the BS process typically follows these stages: EE is followed by cynicism, which results in lower PE. However, from a person-oriented methodology, the manifestation of the BS symptoms would vary across individuals (Mäkikangas and Kinnunen, 2016). In fact, the BS was originally described as a dynamic process resulting from “untreated, long-lasting work stress” (Maslach and Leiter, 2016); that is, a condition where the body is constantly exposed to stressful situations that can lead to BS by means of the homeostasis mechanism alteration. This system has two main components: the sympathetic nervous system and the hypothalamic–pituitary–adrenal (HPA) axis (Foss and Dyrstad, 2011; Gómez-Alcaina et al., 2014). The first one is responsible for preparing the body to respond to a fight-or-flight situation. However, a constantly activated nervous system does not allow the organs to recover, and the person might develop several disorders, including cardiovascular, respiratory, gastrointestinal, muscular, dermatologic, sexual, endocrine, and immune problems (Llaneza Alvarez, 2003; Lever-van Milligen et al., 2020). The second component of the stress system, the HPA axis, becomes activated in the face of EE (Parent-Lamarche and Marchand, 2018); then the cortisol hormone is released (Björntorp, 2001) causing neural, physiological, coronary, and immunological effects in human organism (Lateef et al., 2020). This hormone causes fat accumulation in the abdominal tissue (Kyrou and Tsigos, 2009) and is usually considered to be a biomarker for the BS (Dallman, 2010; Parent-Lamarche and Marchand, 2018; Penz et al., 2018; Penz et al., 2019; Lever-van Milligen et al., 2020).

Additionally, it is known that obesity and metabolic deregulation impair the HPA axis; thus, their relationship is bidirectional. Specifically, obesity and metabolic deregulation influence the HPA axis for different reasons: over activation, a decreased sensitivity to negative feedback mechanisms, or a loss in the sensitivity of peripheral tissues to glucocorticoid activity (Seimon et al., 2013). Another recent meta-analysis has associated stress with BMI (Tenk et al., 2018), and greater abdominal obesity has been associated with a greater HPA responsiveness. Moreover, stressors (psychological and physiological) can also trigger cortisol secretion, thus causing weight gain and, therefore, obesity (Incollingo Rodriguez et al., 2015). All these mechanisms are related to stress by means of the HPA activation. Thus, obesity could increase the sensitivity of physiological stress mechanisms. Some other reasons why stress has an influence on obesity could be that in critical moments (e.g., after a dismissal), people are more likely to start or resume unhealthy habits (e.g., smoking, drinking); they might experience sleep disorders, and they can abruptly modify their eating habits (Nevanperä et al., 2012; Ahola et al., 2012). Similarly, because employees usually have little time to prepare healthy meals, they often resort to unhealthy eating (Barattucci, 2011). Also, stressed managers might forget or skip breakfast (Montiel et al., 2014) or might replace healthy meals with tea and soft and caffeinated drinks (Druce et al., 2004), which increase anxiety, irritability, and exhaustion since they cause sharp rises in blood sugar (Wang et al., 2013). Likewise, a fat-rich diet causes leptin resistance (Carlo et al., 2007). All these factors could be related to the connection between brain and gut, known as brain-gut axis, where it is depicted how microbiomes influence the brain and some psychiatric pathologies associated with emotions and cognition (Bioque et al., 2020).

Therefore, if stress influences the BS and obesity, an association between the two is highly likely. In fact, a more specific research study related to the BS dimensions has shown that EE and cynicism are associated with low physical activity and alcohol consumption, while a reduced perceived PE is associated with obesity (Andreyeva et al., 2014). Therefore, the literature points to the BS and obesity as resulting from the adverse conditions of the work environment (Borak, 2011) and as potentially leading to serious economic and health concerns. Nevertheless, the scientific community has not yet managed to establish a clear relationship between obesity and the BS (Iversen et al., 2012; López-Morales and Garcés, 2012; Nevanperä et al., 2012; Scott and Johnstone, 2012; Solovieva et al., 2013). Hence, considering that the BS and obesity have visible negative effects on employees’ health in both the behavioral and the psychological aspect as well as in the working environment where they interact most of the workday, studying the relationship between both phenomena becomes urgent. However, the literature where a relationship between both conditions is established is scarce, especially in the case of studies considering the effect of individuals’ BMI on the three BS dimensions. One exception is the research carried out by Ahola et al. (2012), who found that only low PE was significantly associated with obesity, while the dimensions of EE and Cynicism were significantly related to low physical activity. In addition, the study of Moueleu Ngalagou et al. (2019), associated BS with low physical activities leisure and sport practice among employees. Additionally, some authors have found evidence of the relationship between the BS and obesity among middle and senior managers of the Mexican manufacturing industry. This is the case of the study by Armenta-Hernández et al. (2018), who studied the relationship between the BS dimensions and BMI by analyzing normal, overweight, and obese individuals. These authors found that the model for managers featuring normal weight has a larger explanatory power than the models developed with overweight and obese employees. Recently, another study (Armenta-Hernandez et al., 2020) found that both BS and physical activity are factors that have a direct effect on the BMI of obese managers, and their research is currently conducting examinations in search for more explicative models. For instance, it was found that PE is related to normal weight because when employees feel efficient at work, they worry about their health, take care of their physical appearance, and even improve their relationships at work (Eberly and Feldman, 2010). Similarly, EE and cynicism have been associated with high BMI since both dimensions can alter eating habits and increase a person’s caloric intake. In fact, eating disorders are usually a result of stress-related emotions (Kouvonen et al., 2005; Nevanperä et al., 2012). Such findings point to BMI as a possible moderator variable, but so far, BMI as a moderator of the BS has been insufficiently studied. Accordingly, a moderator effect of the BMI was thought to be focused on obese individuals.

Recent advances have found relationships between the BS and BMI in obese managers, for example (Armenta-Hernández et al., 2018) and those that relate microbiome, brain and gut activity, (brain-gut axis). Furthermore, the contributions of those authors that have studied the mental disorders such as (Bioque et al., 2020). It is important to test the possible mediating role of BMI in the relationships among the BS dimensions; specifically, the core dimensions (EE and cynicism) with PE, which would be more related to productivity, in the sense that workers with low PE would reduce their motivation and consequently their productivity (Banerjee, 2015). Consequently, this research aims to address this gap by analyzing the relationships within the BS dimensions and the mediating role of BMI, treating the latter as a moderator variable among obese senior and middle managers in the maquiladora industry of Ciudad Juarez.

Specifically, in this study we will test the following hypotheses:

**H1a**: BMI moderates the negative relationship between Emotional Exhaustion and Professional Efficacy in such a way that the relationship is stronger under a high BMI as opposed to under a low BMI.

The tentative hypothesis is that EE is related to PE because the increase in BMI would cause a sensation of fatigue (difficulty of movement, breathless, discomfort), which would, in turn, increase physical exhaustion, affecting emotions and influencing negatively on PE.

**H1b:** BMI moderates the negative relationship between Cynicism and Professional Efficacy since the relationship is stronger under a high BMI as opposed to under a low BMI.

Considering that Cynicism reduces risk perception (i.e., disregarding healthy behaviors such as eating), this tentative hypothesis intends to prove that high cynicism and BMI will negatively influence PE.

### Conceptual Model to Test

**FIGURE 1:**
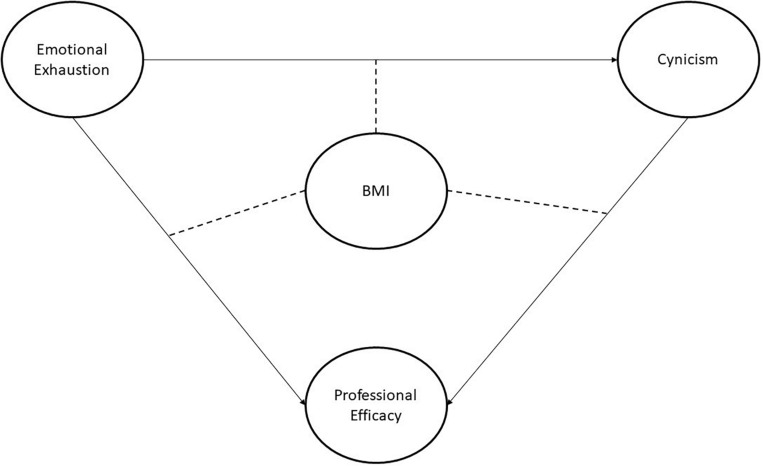
Hypothetical model.

To respond to these hypotheses, the hypothetical model using Partial Least Squares-Structural Equation Modeling (PLS-SEM) will be used. Figure 1 shows the effects of BMI on the relation of the BS dimensions. Note that EE, cynicism, and PE are treated as endogenous latent variables. In turn, EE has five indicators, cynicism has four, and PE has five. On the other hand, BMI is treated as an endogenous latent variable made up of two indicators (weight and height). The latter is a moderator variable, and the relationships are shown as discontinued lines in Figure 1.

## Materials and Methods

### Participants

The data were collected from staff in six maquiladora industries located in Mexico. Three of these companies are automotive manufacturers, two are electrical manufacturing companies, and one assembles miscellaneous products. The sample included senior managers from different departments such as production, human resources, maintenance, environment, and quality. The middle management positions involved in the survey included supervisors, technicians, group leaders, and administrative staff. A total of 361 questionnaires were collected and classified according to the respondent’s BMI values. However, the study considered only the surveys of the 124 respondents classified as obese.

Regarding the sociodemographic characteristics of the complete sample, 69% of the interviewed managers were men, while 31% were women. Additionally, the results showed supervisors and technicians to be the population that suffers most frequently from obesity and overweight. The ages of the people in the sample ranged from 18 to 60 years old, with an arithmetic mean of 37 years and a standard deviation of 9.2 years. Finally, employee seniority ranged from 1 to 372 months, with an arithmetic mean of 103.12 months and a standard deviation of 83.9 months.

The sample included senior and middle managers, namely supervisors, technicians, group leaders, and administrative staff. After each participant signed a consent form, face-to-face interviews were conducted for the MBI-GS. On the other hand, BMI measures were collected by having participants provide their height (m) and weight (kg) measurements (anthropometric variables), and a recommended 2.5 cm was subtracted from each participant’s height values (Kharin and Voloshko, 2011). Nonetheless, once respondents recorded their values, the medical staff (industrial nurses) took such anthropometric measures properly, using the available calibrated equipment provided by each company. These were the measurements that were used in the analyses. The BMI was used as moderator variable, classifying it according to the WHO’s criteria; however, only the obese participants’ data was analyzed; that is, the data of those individuals with a BMI of 30 kg/m^2^ or greater.

### Measures

The Spanish translated version of the Maslach Burnout Inventory-General Survey (MBI-GS) was used to measure the BS. It consists of 16 questions or items grouped into three dimensions: EE, (Emo Exha, five items), cynicism (Cynicism, five items), and PE (Prof_Eff, six items) (Moreno-Jiménez et al., 2001).

Eight field experts, six industrial managers and two scholars, evaluated the survey to make sure the items were properly formulated, considering the research context.

MBI-GS had to be answered using a seven-point Likert scale where: 0 = never, 1 = rarely throughout the year, 2 = sometimes throughout the year, 3 = on many occasions throughout the year, 4 = frequently throughout the year, 5 = almost every day, and 6 = every day.

### Descriptive Analysis of the Sample

The SPSS 24.0^®^ statistical software was used to analyze the sociodemographic information of the obese sample (i.e., gender, level of studies, marital status, and type of contract, seniority, and current position). Additionally, crosstabs were built between variables to better understand the prevalence of obesity and occupational stress among middle and senior managers. Finally, average scores for each dimension, as well as the BS degrees and levels were estimated for the descriptive analysis of the BS.

The statistical procedure used to test the hypotheses of the model followed several steps. First, the SPSS 24.0^®^ statistical software was used to feed the collected data into a database. Then, that database was screened so that missing and extreme values could be identified. Missing values are items that are left unanswered because participants do not know how to answer a question or do not want to respond to it (Ketkar et al., 2012). In this research, the missing values of a survey were replaced with the median value of the item if the missing rate was less than 10%; otherwise, the survey was removed from the analysis (Wold et al., 2001; Lin et al., 2015; Sovilj et al., 2016). As for outliers, every item was standardized, and only those with a value ranging from -4 to 4 were included in the analysis. Finally, absolute values equal to or higher than 4 were considered outliers and replaced by the median (González-Arteaga et al., 2016). The median was used instead of the mean because the data collected was ordinal (Mani et al., 2016). Once these steps were done, SEM required the latent variable validation. This procedure is further explained in the following section.

Once the previous statistical procedure was done and data were screened, the latent variables were validated to determine whether their predictive power to explain the dependent variables was good. This analysis was conducted with the help of the WarPLS software, and the following coefficients were estimated: Cronbach’s alpha and composite reliability, Average Variance Extracted (AVE), Predictive validity indices (Coefficients *R*^2^ and adjusted *R*^2^ were estimated), and Variance Inflation Factors (VIF).

### Model Evaluation

The model was tested using WarpPLS 5.0,^®^ whose main algorithms are based on partial least squares (PLS) – widely recommended for small-sized samples (Braunscheidel and Suresh, 2009). The model’s reliability was tested through a re-sample bootstrapping procedure, which uses randomized samples taken from the original sample in order to improve model stability (Hayes and Preacher, 2010). An important part of the model’s evaluation involved checking for discriminant validity and observing the model efficiency indices. Additionally, direct, indirect, and total effects of significant relationships among variables needed to be determined as well as the effect sizes.

In this research, all the effects were statistically tested for a 95% confidence level (Ibarra and García, 2016; Luque-Reca et al., 2014).

## Results

### Descriptive Information From Obese Participants

Regarding the respondents’ anthropometric information, the minimum height reported was 1.435 m, and the maximum was 1.875 m, with 1.672 m as the arithmetic mean and 0.092 m as the standard deviation. Likewise, the minimum body weight reported was 70 kg, while 115 kg was the maximum. In this case, 92.71 kg was the arithmetic mean, and 11.453 kg was the standard deviation. An additional descriptive analysis in Table 1 revealed that it is more frequent to find obese men than obese women (68.55% vs. 31.45%, respectively).

**TABLE 1:** Descriptive information – obese managers sample.

Variables	*N*	(%)	Variables	*N*	(%)
**Gender**			**Marital status**		
Men	85	68.5	Single	29	23.4
Women	39	31.5	Married	79	63.7
Age (years)			Free union	12	9.7
≤30	16	12.9	Widowed	1	0.8
31–40	43	34.7	Divorced	3	2.4
41–50	55	44.4	Missing data	0	0
≥51	10	8.1	**Worked hours per week**		
Missing data	0	0	32	5	4.0
**Level of studies**			42	28	16.1
Middle school	11	8.9	45	25	20.2
High school	28	22.6	48	46	37.1
Bachelor’s degree	71	57.3	≥56	28	22.6
Graduate degree	12	9.7	Missing data	0	0
Missing data	2	1.6	**Seniority (months)**		
**Type of contract**			≤12	15	12.1
Indefinite	112	90.3	13–24	7	5.6
Temporary	7	5.6	25–48	20	16.1
Other	4	3.2	49–72	8	6.5
Missing data	1	0.8	≥73	74	59.7
			Missing data	0	0
**Current job position**					
Manager	17	13.7			
Supervisor	42	33.9			
Technician	20	16.1			
Group leader	21	16.9			
Manufact. admin.	6	4.8			
Non-Manuf. admin	16	12.9			
Missing data	2	1.6			

### Burnout Syndrome Descriptive Analysis

Burnout Syndrome is a three-dimensional syndrome; therefore, to measure the presence of BS in the obese sample, the average score of each BS dimension was estimated. In scientific research, average scores of BS are usually compared with respect to national average scores. However, national BS scores for Mexico are still unknown. Table 2 shows the average scores of the three BS dimensions: EE, cynicism, and PE.

**TABLE 2:** Average scores of BS in the obese managers sample.

Dimension	Mean
Emotional exhaustion	9.5403
Cynicism	4.9758
Professional efficacy	28.8468

In addition, Table 3 shows the degrees of BS in the obese sample. The results reveal a medium level of BS in EE and cynicism and a low level in PE. It is worth mentioning that the cutoff points of the 33rd and 66th percentile determined the grades.

**TABLE 3:** Degrees of BS for obese managers sample.

Grade	Emotional exhaustion	Cynicism	Professional efficacy
Low	≤6	≤2	<29 **(28.8468)**
Medium	7–11 **(9.5403)**	3–6 **(4.9758)**	29–32
High	>12	>7	>33

*Degree, emotional exhaustion, cynism and professional efficacy values in bold are the actual average values obtained for each BS dimension in the obese managers sample. These values are located in the reference given ranges to determine the degrees of BS for each dimension.*

### Latent Variables Validation

Table 4 presents the latent variable coefficients. According to the R-squared and adjusted R-squared values, all the latent variables have good predictive power to explain the dependent variables. Similarly, based on the Q-squared coefficient values, all the latent variables have predictive validity from a non-parametric perspective. The composite reliability coefficient and the Cronbach’s alpha index also show that all the latent variables have acceptable internal validity. Likewise, the AVE values confirm that there is enough convergent validity. Finally, according to the full collinearity VIFs, none of the latent variables has collinearity problems since the values do not exceed the cut-off value of 3.3. Thus, the model and the independent variables included for this research have good predictive validity.

**TABLE 4:** Validation of latent variables.

Coefficients	Emotional Exhaustion	Cynicism	Professional Efficacy	BMI
R-squared		0.386	0.146	
Adjusted R-squared		0.381	0.132	
Composite reliability	0.943	0.889	0.916	1.000
Cronbach’s alpha	0.924	0.831	0.886	1.000
Average variance extracted	0.766	0.670	0.687	1.000
Full collinearity VIFs	1.634	1.710	1.156	1.000
Q-squared		0.395	0.147	

### Model Evaluation

#### Results of Discriminant Validity Analysis

The results presented by the heterotrait–monotrait ratio of correlations (HTMT) method indicate that all constructs are different from each other. This fact points to good discriminant validity for each variable. Tables 5–7 show the resulting analysis values.

**TABLE 5:** Correlations AVEs.

	**Emotional exhaustion**	**Cynicism**	**Professional efficacy**	**BMI**
Emotional exhaustion	0.875	0.619	–0.241	–0.077
Cynicism	0.619	0.818	–0.300	–0.076
Professional efficacy	–0.241	–0.300	0.808	0.125
BMI	–0.077	–0.076	0.125	1.000

**TABLE 6:** HTMT ratios.

	**Emotional exhausted**	**Cynicism**	**Professional efficacy**
Emotional exhausted			
Cynicism	0.704		
Professional efficacy	0.271	0.358	

**TABLE 7:** Cross-loadings.

	Emotional exhaustion	Cynicism	Professional efficacy
Emotional exhaustion (1)	0.895	0.567	–0.215
Emotional exhaustion (2)	0.892	0.506	–0.168
Emotional exhaustion (3)	0.869	0.547	–0.187
Emotional exhaustion (4)	0.866	0.542	–0.237
Emotional exhaustion (5)	0.854	0.549	–0.250
Cynicism (1)	0.563	0.880	–0.302
Cynicism (2)	0.566	0.901	–0.268
Cynicism (3)	0.478	0.795	–0.244
Cynicism (4)	0.402	0.678	–0.152
Professional efficacy (1)	–0.085	–0.113	0.766
Professional efficacy (2)	–0.192	–0.319	0.829
Professional efficacy (3)	–0.201	–0.178	0.851
Professional efficacy (4)	–0.166	–0.256	0.800
Professional efficacy (5)	–0.327	–0.345	0.794

As can be seen in Table 5, the values in the diagonal are greater than the values shown above and below it. Thus, the square root of the AVE of each construct is higher than the construct’s highest correlation with any other construct in the model.

Table 6 shows the ratio values of the HTMT analysis, whose results indicate a good discriminant validity with respect to the discrimination criteria since they show values lower than 0.85.

Additionally, to confirm the constructs’ discriminant validity, Table 7 includes the values of the cross-loadings of the items used to conform each dimension. Discriminant validity is established when a certain item loading on a construct is higher than all of its correspondent cross-loadings with other constructs. In this case, each item included in a certain construct has the highest value for the loading with its corresponding construct, while all cross-loadings with other constructs are considerably lower. Hence, both the HTMT method and the cross-loading criterion provide evidence of each construct’s discriminant validity for this study.

#### Results of Model Efficiency Indices

**FIGURE 2:**
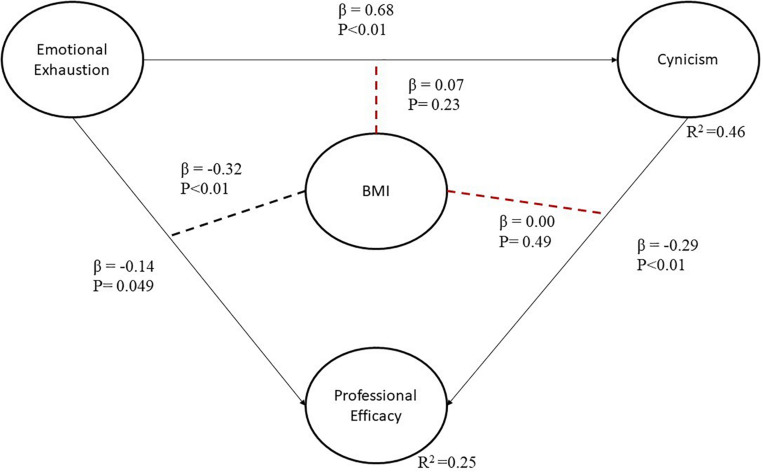
Model with all relationships between latent variables.

Once the latent variables were validated, they were integrated into the model depicted in Figure 2. Note that the figure shows two red discontinued line segments to represent the two moderator effects that are statistically not significant at a 95% confidence level (i.e., the p values that are higher than 0.05). That said, before interpreting the model, it is important to analyze the model efficiency indices. Table 8 explains the correspondent efficiency indicators’ reference values and results.

**TABLE 8:** Model efficiency indices.

Indicators	Value	Decision criteria
Average path coefficient (APC)	0.343	*P* < 0.001
Average R-squared (ARS)	0.320	*P* < 0.001
Average adjusted R-squared (AARS)	0.308	*P* < 0.001
Average blockVIF (AVIF)	3.690	Acceptable if ≤5, ideally ≤3.3
Average full collinearity VIF (AFVIF)	4.077	Acceptable if ≤5, ideally ≤3.3
Tenenhaus (GoF)	0.495	Small ≥0.1, medium ≥0.25, large ≥0.36

The model fit indices shown in Table 8 demonstrate that the model is efficient, since all the *p* values are lower than 0.05. In addition, the results demonstrate that the model has good predictive and explanatory powers, the latter according to the GoF index. In conclusion, the model is efficient.

Once the model was tested and validated through the model fit and quality indices, it was possible to interpret it by analyzing the direct, indirect, and total effects.

**FIGURE 3:**
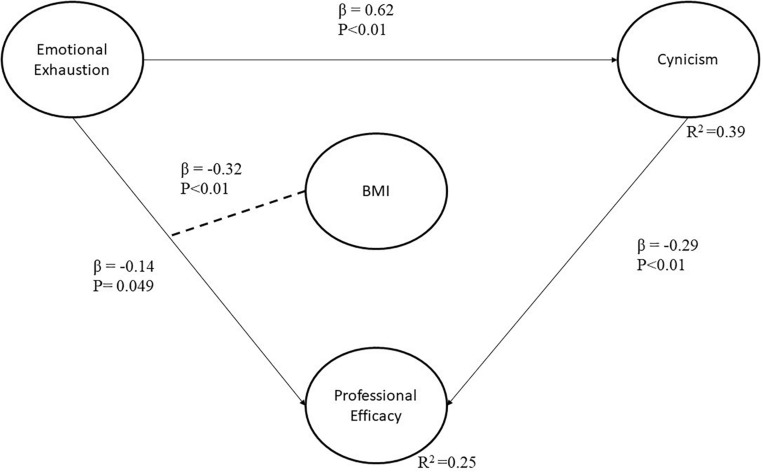
Final model with only significant relationships.

In the model, the two red discontinued line segments in Figure 2 represent the two moderator effects that are statistically not significant at a 95% confidence level. However, the final model, illustrated in Figure 3, has only significant causal relationships.

#### Direct Effects

The direct effects demonstrate why two hypotheses from Figure 2 (i.e., H2 and H3) were removed from the model (i.e., they were not statistically significant at a 95% confidence level). Significant direct effects are shown in black, continuous line segments. All the relationships have a β value and a corresponding p value for the statistical hypothesis test. For instance, in the relationship between EE and PE, β indicates that when the first latent variable increases by one standard deviation, the second latent variable decreases by 0.14 standard deviations or 14%. In the case of BMI as a moderator, the results show that when workers are emotionally exhausted and obese, PE decreases 32%. In other words, if BMI increases as a moderator variable, the strength of the relationship between EE and PE changes. Accordingly, it is to be noted that PE had a negative value of 0.14 before the moderator effect, but the value increased to 0.32 when BMI was included in the relationship. The remaining relationships in the model can be similarly interpreted.

Based on the standardized values of the parameters in Figure 3, the following equations can be formulated:

C⁢y⁢n⁢i⁢c⁢i⁢s⁢m=0.62⁢x⁢e⁢m⁢o⁢t⁢i⁢o⁢n⁢a⁢l⁢e⁢x⁢h⁢a⁢u⁢s⁢t⁢i⁢o⁢n+e⁢r⁢r⁢o⁢r

P⁢r⁢o⁢f⁢e⁢s⁢s⁢i⁢o⁢n⁢a⁢l⁢e⁢f⁢f⁢i⁢c⁢a⁢c⁢y=0.14⁢x⁢e⁢m⁢o⁢t⁢i⁢o⁢n⁢a⁢l⁢e⁢x⁢h⁢a⁢u⁢s⁢t⁢i⁢o⁢n+0.29⁢x

c⁢y⁢n⁢i⁢c⁢i⁢s⁢m+0.32⁢x⁢(B⁢M⁢I⁢x⁢e⁢m⁢o⁢t⁢i⁢o⁢n⁢a⁢l⁢e⁢x⁢h⁢a⁢u⁢s⁢t⁢i⁢o⁢n)+e⁢r⁢r⁢o⁢r

Every dependent latent variable has an *R*^2^ value. This value indicates the percentage of variance explained by independent latent variables. The results in Table 9 show that three independent latent variables contribute to the variance of PE, whose *R*^2^ value equals 0.25; where EE explains 4.3%, cynicism explains 10.4%, and BMI × emotional exhaustion explains 10.7%. In addition, EE explains up to 38.6% of the variability of Cynicism. This analysis can be appreciated in Table 7.

**TABLE 9:** Contribution to the R-squared.

	Emotional exhaustion	Cynicism	BMI × emotional exhaustion	*R*^2^
Cynicism	0.386			0.386
Professional Efficacy	0.043	0.104	0.107	0.25

Concerning the direct effects among the BS dimensions, the direct effect between EE and cynicism was found to be the largest since when the EE variable increased by one unit, the cynicism variable increased by 0.62 units. The second largest effect observed was the direct effect between cynicism and professional performance: when the cynicism variable increased by one unit the PE variable decreased by 0.29 units.

#### Indirect Effects

Latent variables can have indirect effects on other variables through different segments. In this model, there is only one statistically significant indirect effect, that of EE on PE (*P* < 0.001; β = -0.162). In other words, when EE increases by one standard deviation, PE increases by 0.162 standard deviations. As for the effect sizes, EE explains 4.3% of the variance of PE (since *R*^2^ = 0.043) through cynicism.

The model presents a unique indirect effect among the dimensions of BS, namely the relationship between the EE variable and the PE variable through the cynicism variable, where the EE variable contributes with -0.13 units to the decrease in PE.

#### Total Effects

Table 10 shows the sum of the direct and indirect effects of every relationship. Each parameter includes a p value to determine the statistical significance of the effects. Similarly, the table includes the effect sizes. Note that all the total effects are statistically significant at a 95% confidence level since all the p values are lower than 0.05. As for the relationship between BMI and PE, it was concluded that when the first latent variable increases by one standard deviation, the second latent variable decreases by 0.320 standard deviations. Likewise, the percentage of variance explained by the independent variable is 10.7%. All the relationships can be similarly interpreted.

**TABLE 10:** Total effects between latent variables.

	Emotional Exhaustion	Cynicism	BMI
Cynicism	β = 0.621 (*P* < 0.001)		
	ES = 0.386		
Professional efficacy	β = 0.322 (*P* < 0.001)	β = −0.29 (*P* < 0.001)	β = −0.320 (*P* < 0.001)
	ES = 0.095	ES = 0.104	ES = 0.107

## Discussion

This study aimed to test the relationships between the BS dimensions, specifically that of EE and Cynicism with PE, factoring in the mediating role of BMI. First the relationships between these dimensions without the mediating role of BMI were explored. The results indicated that EE and Cynicism are related to PE, thus confirming the first two hypotheses (Leiter and Maslach, 1988; Shirom et al., 2006). Therefore, our study contributes to the understanding of the relationship among the BS dimensions by concluding that dimensions are dependent and are part of the same phenomena (Kim and Ji, 2009). Hence, EE and Cynicism reduce PE, which would lead to a reduction in productivity.

Once the relationship between the BS dimensions was tested, the next step was to investigate the mediating role of BMI on such relationships. The results support both hypotheses, by showing that BMI does exert a role in the relation between EE, Cynicism, and PE. In such a relationship, the higher the EE and BMI, the greater the negative influence on PE. These results are consistent with the theory that BMI is related to the BS dimensions as has been shown before (Ahola et al., 2012; Armenta-Hernández et al., 2018). These results are also in line with the findings in previous research works, where EE has appeared as the main BS dimension associated with BMI. For instance, Nevanperä concluded that burned out employees tend to eat uncontrollably because of negative emotions (Nevanperä et al., 2012) and EE (Kouvonen et al., 2005). Moreover, this study is consistent with Luckhaupt et al. (2014), who concluded that office workers, administrative staff, architects, and engineers are more likely to suffer from overweight and obesity (Luckhaupt et al., 2014). On the other hand, according to Luque-Reca et al., middle managers suffer from greater levels of perceived stress than senior managers do since they work more hours and must follow their superiors’ orders (Luque-Reca et al., 2014).

Armenta-Hernández et al. (2018), associated the dimensions of the BS with the different BMI classifications, namely normal weight, overweight and obesity, by showing EE and Cynicism as the dimensions with the highest indirect effect on BMI (Armenta-Hernández et al., 2018). They also mention the need to consider other factors such as physical activity and eating habits to better describe obesity and overweight. Our results are also consistent with those of other studies. For example, Ahola found that low Professional Efficiency is directly related to obesity (Ahola et al., 2012). Nevanperä et al. (2012), show the fact that EE is related to uncontrolled eating and emotions in the working population, while Proper et al. (2013), address the changes in BMI. Furthermore, physiologically speaking, the stress system is activated by EE, which is, in turn, linked to the release of cortisol, the hormone responsible for obesity (Gómez-Alcaina et al., 2014; Epel et al., 2001; Kyrou and Tsigos, 2009; Foss and Dyrstad, 2011; Marchand et al., 2014; Newman et al., 2007; Torres and Nowson, 2007).

Consequently, both hypotheses are proven. As was indicated in the introduction, both hypotheses were tentative, considering that although the literature suggested a relationship between these variables, the direction or the nature is not clear. From this study’s point of view, a possible explanation could be that people with high EE and Cynicism and a high BMI will increase their probability of feeling less PE. These results are interesting, but from the standpoint of this research, they are but the surface of a highly complex relation.

A tentative explanation would be that the increase in BMI would increase fatigue sensation (difficulty of movement, breathless, among others), which would, in turn, increase physical exhaustion, affecting emotions and causing a negative influence on PE. However, EE and BMI could be bidirectionally related, making the main (or first) factor difficult to elucidate. The same explanation could apply to Cynicism. Hence, more studies would be necessary to understand the nature and the direction of such relationships with PE.

Another important issue is the explanation of why BMI mediates in the relation between EE and Cynicism and PE. From a behavioral point of view, both BS dimensions could modify the eating behavior habits resulting from anxiety and could cause risky behaviors (unhealthy eating) as it has been reported to happen when people suffer from a highly stressful situation. Complementarily, these behaviors could modify the microbiome, depending on the type of food (usually not healthy). Thus, if stress increases the risk of unhealthy eating and the release of cortisol, both factors would modify the microbiome, which is associated with brain changes that affect emotions. This, then, could be the way the BS dimensions and BMI could be interacting, causing difficulties in regulating emotions and increasing the probability of BS.

Nonetheless, while the above is a plausible explanation, more controlled studies are needed to explain the nature of these findings. However, the authors consider this an important topic that could be addressed in future studies, and which could lead to new insights from the organizational point of view. That is, the BS appears to be a complex syndrome that depends not only on psychosocial variables but on other variables such as BMI. This future research could explain the relationships between psychological factors (stress and burnout) with obesity and other metabolic syndromes.

In short, this paper leaves different questions unanswered, which are worth addressing. The first one relates to the matter of how the different BS dimensions are related to each other and how the BMI variable mediates in such a relationship. Secondly, the extent to which Microbiome-Gut-Brain Axis (Bioque et al., 2020) is related to BS along with other mental illnesses, including how stress-related hormones are related to inflammatory markers, should be explored, as well as the way such an interaction may affect mood disorders (Schatzberg, 2015; Soria et al., 2018).

## Conclusion

This work offers several contributions. First, it describes the relationship between the three BS dimensions and BMI among obese managers, demonstrating how one important variable that would affect work productivity (PE) (Maslach and Jackson, 1981) is affected by both conditions. In addition, the structural equation model proposed shows this behavior in standard deviations to help maquiladora companies identify, and eventually prevent and reduce, BS incidence and its negative effects, one of which is BMI. In fact, targeting BS and BS-related problems can prevent economic losses derived from employees’ poor performance and health issues. This research support Mexican maquiladora industry in their efforts toward a healthier work environment, by providing relevant knowledge on the effects of obesity on their employees’ PE.

## Industrial Implications

The BS and obesity have serious industrial implications. Data on the prevalence of these conditions are widely available for developed countries; therefore, these nations have been able to take corrective actions to prevent and manage the incidence of obesity and the BS in the workplace (Jalali et al., 2019). However, in developing countries such as Mexico, the scientific community still needs to explore the occurrence of these conditions, their relationship, and the implications of such relationships for the industrial sector. Studies on BMI, obesity and BS in developing countries are often exclusive to some professions, and the information sources are unknown. Additionally, obesity is one of the main public health problems in Mexico. In this research, BMI as a moderator variable is associated with the three-burnout dimensions among obese individuals to measure and analyze the effects of the resulting relationships on work performance. From this perspective, this work promotes and contributes to the study of both obesity and the BS in Mexico when the moderator effect of BMI intervenes, highlighting the importance of controlling psychosocial factors at work, particularly work stress. We propose that organizations could prevent obesity by controlling the weight of their workers and reducing the existence of unhealthy food in the workplace. Complementarily, reducing EE and Cynicism by means of preventive programs would improve the sense of personal efficacy and therefore increasing productivity. In developing countries, encouraging research on obesity and the BS, as well as on the moderator effect of BMI, can have positive implications for those industries seeking to improve their performance by taking into consideration the employees’ quality of life. This type of research can also help industries to better understand the magnitude of the problem and develop efficient organizational strategies to prevent occupational stress and public health problems.

## Data Availability Statement

The datasets generated for this study are available on request to the corresponding author.

## Ethics Statement

The studies involving human participants were reviewed and approved by Institutional Ethics and Bioethics Committe of the Autonomous University of Ciudad Juarez-Veredict Number CIEB-2019-43. The patients/participants provided their written informed consent to participate in this study. Written informed consent was obtained from the individual(s) for the publication of any potentially identifiable images or data included in this article.

## Author Contributions

OA-H, AM-M, and MC-A performed the data collection. OA-H and AM-M wrote the manuscript. AM-M acquired the funding. OA-H and MC-A analyzed the model. MS-R contributed to the models’ psychophysiological understanding. YB-L reviewed the statistical procedure. CB-A improved the writing and readability of the manuscript. All authors contributed to the article and approved the submitted version.

## Conflict of Interest

The authors declare that the research was conducted in the absence of any commercial or financial relationships that could be construed as a potential conflict of interest.
